# C-reactive protein is differentially modulated by co-existing infections, vitamin deficiencies and maternal factors in pregnant and lactating indigenous Panamanian women

**DOI:** 10.1186/s40249-017-0307-1

**Published:** 2017-06-02

**Authors:** Doris González-Fernández, Emérita del Carmen Pons, Delfina Rueda, Odalis Teresa Sinisterra, Enrique Murillo, Marilyn E. Scott, Kristine G. Koski

**Affiliations:** 1 School of Dietetics and Human Nutrition, Macdonald Campus of McGill University, 21,111 Lakeshore Road, Ste-Anne-de-Bellevue, QC H9X 3V9 Canada; 2 Department of Nutritional Health, Ministry of Health, Ancón, Panama City, Panama; 3 “Comarca Ngäbe-Buglé” Health Region, Ministry of Health, San Félix, Chiriquí Province Panama; 4 “Panamá Norte” Health Region, Ministry of Health, Las Cumbres Square, Transithmian Highway, Panama City, Panama; 5 Department of Biochemistry, University of Panama, Simón Bolivar Avenue (Transithmian Highway), Panama City, Panama; 6 Department of Biochemistry, University of Panama, Manuel Espinoza Batista and Jose De Fabrega Avenues, Panama City, Panama; 7 Institute of Parasitology and Centre for Host-Parasite Interactions, Macdonald Campus of McGill University, 21,111 Lakeshore Road, Ste-Anne-de-Bellevue, QC H9X 3 V9 Canada; 8 School of Dietetics and Human Nutrition and Centre for Host-Parasite Interactions, Macdonald Campus, McGill University, 21,111 Lakeshore Road, Ste-Anne-de-Bellevue, QC H9X 3 V9 Canada

**Keywords:** CRP, Intestinal parasites, Caries, Vaginal infections, Folic acid deficiency, Wood smoke, Neutrophil/lymphocyte ratio, Plateletcrit, Pregnancy, Lactation

## Abstract

**Background:**

The usefulness of C-reactive protein (CRP) as a non-specific marker of inflammation during pregnancy and lactation is unclear in impoverished populations where co-existing infections and vitamin deficiencies are common.

**Methods:**

This cross-sectional study in Panama recruited 120 pregnant and 99 lactating Ngäbe-Buglé women from 14 communities in rural Panama. Obstetric history, indoor wood smoke exposure, fieldwork, BMI, vitamins A, B_12_, D, and folic acid, and inflammation markers (CRP, neutrophil/lymphocyte ratio (NLR), plateletcrit and cytokines) were measured. Multiple regressions explored both associations of CRP with other inflammatory markers and associations of CRP and elevated CRP based on trimester-specific cut-offs with maternal factors, infections and vitamin deficiencies.

**Results:**

CRP was higher in pregnancy (51.4 ± 4.7 nmol/L) than lactation (27.8 ± 3.5 nmol/L) and was elevated above trimester specific cut-offs in 21% of pregnant and 30% of lactating women. Vitamin deficiencies were common (vitamin A 29.6%; vitamin D 68.5%; vitamin B_12_ 68%; folic acid 25.5%) and over 50% of women had two or more concurrent deficiencies as well as multiple infections. Multiple regression models highlighted differences in variables associated with CRP between pregnancy and lactation. In pregnancy, CRP was positively associated with greater indoor wood smoke exposure, caries and hookworm and negatively associated with *Ascaris* and vaginal *Lactobacillus* and *Bacteroides/Gardnerella* scores. Consistent with this, greater wood smoke exposure, caries as well as higher diplococcal infection score increased the odds of trimester-elevated CRP concentrations whereas longer gestational age lowered the likelihood of a trimester-elevated CRP. During lactation, folic acid deficiency was associated with higher CRP whereas parity, number of eosinophils and *Mobiluncus* score were associated with lower CRP. Also, a higher BMI and *Trichomonas vaginalis* score increased the likelihood of an elevated CRP whereas higher parity and number of eosinophils were associated with lower likelihood of an elevated CRP.

**Conclusions:**

Infections both raise and lower CRP concentrations in pregnant and lactating mothers. Only folic acid deficiency during lactation was associated with higher CRP concentrations. Caution is required when interpreting CRP concentrations in pregnant and lactating women who have co-existing nutrient deficiencies and multiple infections.

**Electronic supplementary material:**

The online version of this article (doi:10.1186/s40249-017-0307-1) contains supplementary material, which is available to authorized users.

## Multilingual abstracts

Please see Additional file 1 for translations of the six official working languages of the United Nations.

## Background

C-reactive protein (CRP) is widely used in clinical practice as a non-specific acute-phase indicator of inflammation [1], given its rapid production by the liver and release into circulation [2] and its stimulation by several cytokines including IL6, IL1β and TNFα [3]. CRP also helps the body recognize the presence and severity of infections [4–6] and is used as an indicator of low-grade inflammation in chronic infections [7, 8] and chronic diseases [9]. In addition, CRP responds to a variety of maternal factors. Multiparity [10, 11] and exposure to wood smoke [12, 13] have been associated with elevated CRP in pregnancy and/or lactation whereas recreational exercise has been shown to decrease CRP [14, 15]. CRP normally increases during gestation [16], returning to basal concentrations shortly after delivery [17]. A recent study in a population of indigenous Australian women showed CRP concentrations differed by trimester [18]. Previously, some authors have recommended use of trimester-specific cut-offs to define elevated concentrations of CRP during pregnancy [19, 20] whereas other authors have tried to identify cut-offs for predicting adverse pregnancy outcomes such as gestational diabetes and choriamnionitis [21].

CRP is modulated by nutritional status and deficiencies of vitamins A [22], D [23] and folic acid [24], but several studies have reported no association of CRP with vitamin B_12_ [25, 26]. CRP reportedly is higher in obese pregnant women [27], but this association was lost by the end of pregnancy [28]. Folic acid concentration has been negatively correlated with CRP in pregnant women from Korea [24] and in a non-pregnant, non-lactating population in the US [25]. Also, serum vitamin A concentrations were negatively correlated with CRP in pregnant women from Guinea-Bissau [29] and Ethiopia [22] and in lactating women from Mali [30]. Vitamin D supplementation was reported to decrease CRP concentrations in healthy Iranian pregnant women [31] but not in African-American women in their second trimester [32]. However, a large study with pregnant women in Nepal showed no effect of multiple micronutrient supplementation on indicators of inflammation including CRP [33].

CRP is a well-known soluble pattern recognition molecule that responds to infections [34]. The higher elevation of CRP in the presence of combined viral and bacterial pneumonia [35], combined human immunodeficiency virus (HIV) and tuberculosis [36], and combined dengue and malaria [37] compared with the presence of single infections, highlights the likelihood that co-occurring chronic infections are likely to cumulatively increase concentrations of CRP. On the other hand, evidence suggests that helminth infections can lower inflammation [38].

In developing countries where biomarkers of inflammation are not always available in remote settings, CRP is a widely available and cost-effective marker of inflammation [6] that can be used alongside the neutrophil/lymphocyte ratio (NLR) and platelet indices as predictors of adverse pregnancy outcomes [39–41]. Our previous work in an indigenous population living in conditions of extreme poverty in Panama revealed not only a range of bacterial, fungal, protozoan, helminth, and ectoparasite infections among pregnant and lactating women but also synergistic and antagonistic interactions among the various pathogens [42]. Furthermore, informal conversations with physicians highlighted the challenge they faced in relying on CRP as a marker of inflammation under these conditions. This led us to investigate whether CRP may respond to mild-moderate infections and/or micronutrient deficiencies experienced by pregnant or lactating women.

Our specific objectives were (1) to record CRP concentrations in a population of indigenous pregnant and lactating women and (2) to assess which among a range of other inflammatory markers (platelets, neutrophils, cytokines), environmental factors (wood smoke, field work), infections (oral, urogenital, intestinal, skin), and micronutrient deficiencies (folate, vitamins A, B_12_ and D) were associated with CRP concentrations during pregnancy and lactation.

## Methods

### Design

A detailed description of the study design and methodology and inclusion/exclusion criteria was previously published [42]. Briefly, this cross sectional study included 120 pregnant women (11 first, 43 second and 66 third trimester) and 99 lactating women belonging to the Ngäbe-Buglé community in the Western rural region of Panama and was conducted between August and December 2010. Questionnaires and clinical exams were conducted, and venous blood, urine, vaginal and faecal samples were obtained.

### Anthropometry, obstetric history, environmental factors

Weight and height were recorded. Body mass index (BMI) [weight/(height)^2^] was calculated and was corrected in pregnant women by subtracting estimated fetal weight using Johnson’s formula [43]. Pregnant women were classified individually as underweight, normal weight or overweight/obese according to the Pan-American Health Organization weight-for-height chart [44]. The BMI for lactating women was classified as for the general population: underweight (BMI < 18.5 kg/m^2^), normal weight (BMI between 18.5 and 24.9 kg/m^2^), overweight and obese (BMI ≥ 25 kg/m^2^) [45]. Participants answered questions on obstetric history, hours of indoor wood smoke exposure and fieldwork hours per day.

### C Reactive protein

CRP was processed at the Central Reference Laboratory in Public Health of the Gorgas Memorial Institute for Health Studies in Panama City in duplicate using solid phase enzyme-linked immunosorbent assay (MP Biomedicals, Orangeburg, NY), with a minimum detectable concentration of 0.9 nmol/L. The cut-offs for elevated CRP were set at 193.3 nmol/L and 77.1 nmol/L in the second and third trimesters, respectively [20]. During the first trimester and during lactation, the cut-off for non-pregnant women of 28.5 nmol/L was used [20].

### Infections and co-morbidities

Oral, skin and urogenital infections were assessed clinically. Urine (118 pregnant and 94 lactating women), vaginal (119 pregnant and 79 lactating women) and faecal samples (120 pregnant and 23 lactating women) were analysed for infections as previously described [42]. Presence or absence of caries, scabies, asymptomatic bacteriuria/urinary tract infection (AB/UTI), *Ascaris*, hookworm and *Trichuris* were recorded and semi-quantitative scores (0 to 4) of vaginal *Lactobacillus*, *Bacteroides/Gardnerella*, *Mobiluncus*, *Trichomonas vaginalis*, yeast and diplococcal infections were recorded. Bacterial vaginosis (BV) was diagnosed using the Nugent score, calculated as: *Bacteroides/Gardnerella* score + (4 – *Lactobacillus* score) + (*Mobiluncus* score/2) [46]. HIV and gestational diabetes were ruled out; the study area was non-endemic for malaria. Mothers reported no allergic conditions or history or symptoms of chronic cardiovascular, renal or autoimmune diseases.

### Micronutrient concentrations

Serum samples were analysed for folic acid and vitamin B_12_ concentrations using immunoelectro-chemiluminescence on the analyser MODULAR E170 (Roche Diagnostics GmBH, Mannheim, Germany) at the Canadian Diagnostics Laboratory, a national reference laboratory in Montreal. Folic acid deficiency was defined as < 10 nmol/L and vitamin B_12_ deficiency as < 150 pmol/L [47].

Serum vitamin A was detected using high-performance liquid chromatography at the Institute of Scientific Research and High Technology Services-INDICASAT in Panama City [48]. The cut-off for vitamin A deficiency was set at < 1.05 μmol/L during pregnancy and lactation [49], and the cut-off for higher than normal vitamin A was set at > 1.5 μmol/L for both pregnancy [20] and lactation [50].

Serum OH vitamin D was assayed using the LIAISON, DiaSorin direct competitive chemiluminescence immunoassay (lot 125756, kit 72947). The intra-assay coefficients of variation were 1.2% and 3.3% for pregnant women and 2.2% and 3.1% for lactating women. Respective inter-assay variabilities were 3.8% and 17.1%. We used a cut-off for vitamin D deficiency of < 50 nmol/L [51].

### White blood cells (WBC) and platelets

Blood samples were analysed for complete blood cell count using a BC-5500 Mindray Auto Hematology Analyzer. Data were recorded on total white blood cells, number of neutrophils, lymphocytes, eosinophils and basophils, NLR, and number of platelets (120 pregnant and 99 lactating women). Plateletcrit, mean platelet volume (MPV) and platelet distribution width (PDW) were also recorded.

Concentrations of cytokines (Interleukin (IL) 1β, IL4, IL6, IL10, IL12, IL13, IL17, TNFα and INFγ) were analyzed via Luminex (Luminex Corp., U.S.A.) as part the Human 10-plex Cytokine/Chemokine Magnetic Bead Panel (Cat. HCYTOMAG-60 K; Millipore Corporation Canada) according to manufacturer instructions at the School of Dietetics and Human Nutrition, McGill University. Minimum detection limits (pg/mL) were: IL1β = 0.8, IL4 = 4.5, IL6 = 0.9, IL10 = 1.1, IL12 = 0.6, IL13 = 1.3, IL17 = 0.7, TNFα = 0.7, INFγ = 0.8. For each essay, standards and quality controls were analysed in duplicate and quality controls were within accepted ranges.

### Statistical analyses

Statistics were performed using STATA 14 (StataCorp LP, Texas, USA). CRP was not normally distributed and was log transformed unless otherwise indicated. Kolmogorov-Smirnov test compared those non-normally distributed variables (age, parity, wood smoke, BMI and interleukins) between pregnancy and lactation. Student’s *t*-tests compared normally distributed vitamin D, MPV, PDW and plateletcrit, and log transformed folic acid, vitamin B_12_, vitamin A, WBC counts and total platelets between pregnancy and lactation. Chi^2^ analysis compared the frequency of vitamin deficiencies between pregnancy and lactation. T-tests were also used to compare log CRP between binary classifications of exposure (yes/no) to wood smoke and fieldwork, presence/absence of specific infectious and micronutrient deficiencies in both pregnant and lactating women. One-way ANOVA compared log CRP by trimesters, by age (<19 yrs, 19 – 35 yrs and > 35 yrs), by parity (1, 1 – 4 and ≥ 5 pregnancies) and by BMI (underweight, normal weight, overweight) classifications. Spearman correlations were used to identify correlations of non-transformed CRP with WBC, platelet indices and cytokines and Kruskal Wallis test to compare pregnant and lactating women with 0, 1, 2, or 3 concurrent micronutrient deficiencies. *P*-values < 0.05 were considered significant. A multiple linear regression analysis associated inflammatory markers (WBC, platelet and cytokines) with log-transformed CRP in pregnancy and in lactation.

Separate stepwise multiple linear regression models for log CRP and multiple logistic regression models for elevated CRP were done for both pregnant and lactating women, controlling for parity and BMI as well as gestational age of pregnant women and weeks post-partum for lactating women. In the final linear regression model for log CRP and logistic regression model for elevated CRP, we included environmental factors, infections with a prevalence > 10%, and vitamin deficiencies. The following variables were entered: hours of wood smoke exposure and fieldwork; presence of caries, semi-quantitative scores of vaginal *Lactobacillus*, *Bacteroides/Gardnerella*, *Mobiluncus*, *T. vaginalis*, yeast and diplococcal infections and deficiencies of folic acid, vitamins B_12_, A and D. In pregnancy, presence of *Ascaris*, hookworm and *Trichuris* was also included. In lactation, eosinophil count was included.

All models were tested for collinearity using variance inflation factors (VIF) and the stability of the regression coefficients was assessed using the condition number. Models were considered suitable if VIF < 10 and condition number < 30. AB/UTI and scabies were excluded from models of lactating women due to collinearity.

## Results

### Maternal characteristics and infections

The population was characterized by high rates of adolescent pregnancy (26.9%), grand-multiparity (27.4%), and indoor wood smoke exposure (94.5%). The majority of women (62.1%) were of normal weight, 6.8% were underweight and 31% of women were overweight. About half of the women (46.1%) worked in the field. Almost all women (97%) were infected with at least one vaginal pathogen. Other common infections included hookworm (55.2%), AB/UTI (45.7%), *Ascaris* (30%), caries (19.6%), scabies (15.5%) and *Trichuris* (11.9%), as reported previously [42].

### Vitamins

**Fig. 1:**
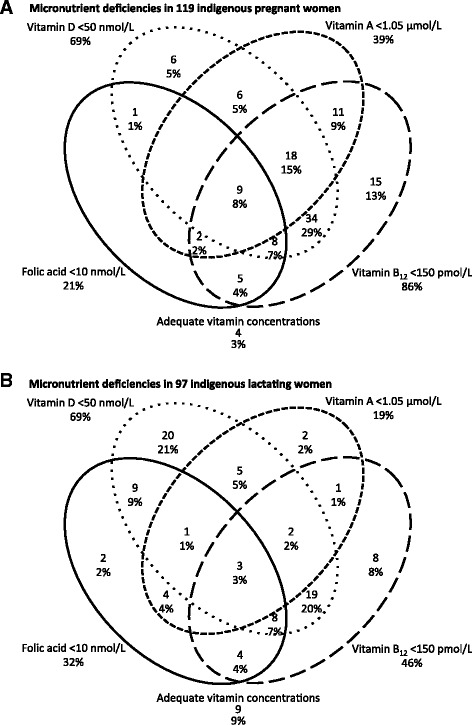
Venn diagram of co-existing micronutrient deficiencies in **a** pregnant women, and **b** lactating women. Legend: In both pregnancy and lactation, ovals drawn with a continuous line represent women with folic acid < 10 nmol/L, ovals with large-dashed lines represent women with vitamin B_12_ < 150 pmol/L, medium-dashed lines circumscribe women with vitamin A < 1.05 μmol/L and dots denote women with vitamin D < 50 nmol/L. Only 3% of pregnant and 9% of lactating women had adequate vitamin concentrations

Deficiencies of vitamin B_12_ (85.8% in pregnancy; 46% in lactation, *χ* ^2^ test *P* < 0.0001) and vitamin A (38.6% in pregnancy; 18.5% in lactation, *χ* ^2^ test *P* = 0.001) were more common in pregnancy. Neither deficiency of vitamin D (68.3% in pregnancy; 68.7% in lactation, *χ* ^2^ test *P* = 0.95) nor folic acid (31.3% in pregnancy; 20.8% in lactation, *P* = 0.07) differed between pregnancy and lactation. Concurrent micronutrient deficiencies were more often found in pregnant than in lactating women (Kruskal-Wallis test: *P* = 0.0003) (Fig. 1). Two concurrent deficiencies were found in 48% of pregnant and 43.3% of lactating women, 3 concurrent deficiencies occurred in 23.5% of pregnant and 11.3% lactating women, and 4 concurrent deficiencies were found in 7.5% of pregnant and 3.1% of lactating women. *T*-test comparisons of vitamin concentrations revealed that pregnant women had higher folic acid concentrations but lactating women had higher concentrations of vitamins A and B_12_ (Table 1).

**Table 1:** Comparisons between impoverished pregnant and lactating women in rural Panama^1^

	Pregnancy	Lactation	Pregnancy	Lactation
	Mean ± *SE*	Mean ± *SE*	Median (IQR)^2^	Median (IQR)^2^
Maternal Characteristics				
Age, y	24.8 ± 0.6	25 ± 0.7	24 (11)	23 (10)
Parity, #	3.4 ± 0.2	3.5 ± 0.2	3 (4)	3 (3)
Wood smoke, h/d	2.5 ± 0.1	2.3 ± 0.1	2 (1)	2 (1)
Fieldwork, h/d	4.5 ± 0.3	4.7 ± 0.3	4 (3.5)	4 (4)
BMI (kg/m^2^)	25.1 ± 0.3	25.5 ± 0.3	24.7 (4.6)	25.1 (4.9)
Nutritional Status Indicators				
Folic acid, nmol/L	16.0 ± 0.7^a*^	13.5 ± 0.6^b^	14 (8.1)	11.9 (7.3)
Vitamin B_12_, pmol/L	111.1 ± 4.3^b^	170.2 ± 7.2^a*^	100 (48)	153 (62)
Vitamin A, μmol/L	1.20 ± 0.03^b^	1.47 ± 0.04^a**^	1.17 (0.46)	1.42 (0.66)
Vitamin D, nmol/L	43.1 ± 1.4	43.0 ± 1.4	39.7 (22.0)	42.2 (19.8)
White Blood Cells (WBC)				
Total WBC × 10^9^/L	8.92 ± 0.21^a*^	8.21 ± 0.18^b^	8.6 (2.8)	7.8 (2.4)
Neutrophils × 10^9^/L	6.07 ± 0.20^a**^	4.55 ± 0.15^b^	5.9 (2.3)	4.1 (1.7)
Lymphocytes × 10^9^/L	2.01 ± 0.04^b^	2.45 ± 0.06^a**^	1.9 (0.6)	2.3 (0.8)
Monocytes × 10^9^/L	0.39 ± 0.01	0.38 ± 0.01	0.37 (0.14)	0.36 (0.12)
Eosinophils × 10^9^/L	0.39 ± 0.02^b^	0.78 ± 0.06^a**^	0.36 (0.29)	0.61 (0.57)
Basophils × 10^9^/L	0.03 ± 0.00^b^	0.05 ± 0.00^a**^	0.03 (0.02)	0.04 (0.03)
Neutrophil/lymphocyte ratio (NLR)	3.15 ± 0.14^a**^	1.96 ± 0.08^b^	2.9 (1.1)	1.7 (0.9)
Platelets				
Total platelets × 10^9^/L	262.8 ± 5.6^b^	323.4 ± 1.0^a**^	262 (82.5)	306 (113)
MPV, fL	8.9 ± 0.1	8.8 ± 0.10	8.8 (1.2)	8.7 (1.4)
PDW	15.9 ± 0.03^a**^	15.6 ± 0.04^b^	15.9 (0.6)	15.6 (0.5)
Plateletcrit, %	23.5 ± 0.4^b^	28.3 ± 0.71^a**^	23 (7)	26.9 (8)
Cytokines, pg/mL				
IL1-B	4.9 ± 0.5^a**^	1.5 ± 0.4^b^	1.72 (7.7)	0.02 (1.3)
IL4	19.7 ± 2.5^a**^	3.9 ± 0.6^b^	9.1 (23.4)	2.1 (4.0)
IL6	8.8 ± 1.1^a**^	4.9 ± 1.2^b^	1.6 (11.8)	1.6 (0)
IL10	3.9 ± 0.6^a**^	1.3 ± 0.3^b^	1.2 (4.8)	0.3 (1.2)
IL12	15.6 ± 3.1^a*^	4.6 ± 0.8^b^	1.5 (21.5)	1.3 (5.7)
IL13	4.4 ± 0.6^a**^	1.2 ± 0.2^b^	1.6 (7.4)	0.9 (0.9)
IL17	6.5 ± 0.7^a**^	3.5 ± 1.2^b^	2.3 (10.9)	0.8 (1.2)
INF-γ	9.0 ± 1.0^a**^	7.6 ± 2.6^b^	3.6 (13.2)	1.6 (3.4)
TNF-α	7.5 ± 0.7^a*^	6.0 ± 0.5^b^	6.5 (12.4)	4.4 (6.9)
CRP (nmol/L)	51.6 ± 4.7^a**^	27.6 ± 3.5^b^	32.8 (58.1)	12.3 (30.4)

^1^Values are means ± *SE* or IQR, *n* = 120 for pregnancy with the exception of wood smoke (114), fieldwork (60), vitamin A and cytokines (119), MPV, PDW and plateletcrit (115). For lactation, *n* = 99 with the exception of wood smoke (93), fieldwork (41), vitamin A and MPV, PDW and plateletcrit (97). Means with different letter superscripts are significantly different at *P < 0.05 and **P < 0.0001

^2^IQR = Q3 – Q1 value

### Inflammation markers

The numbers of WBC and neutrophils, the NLR, and PDW were higher in pregnant women, but numbers of lymphocytes, eosinophils, basophils, platelets and plateletcrit were higher in lactating women (Table 1). Concentrations of all measured cytokines (IL1β, IL4, IL6, IL10, IL12, IL13, IL17, INFγ, TNFα) were higher in pregnant than in lactating women (Table 1).

### CRP

**Fig. 2:**
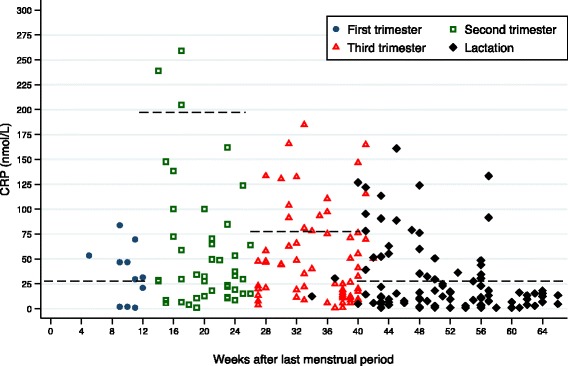
Mean CRP (nmol/L) by weeks after last menstrual period in indigenous pregnant and lactating women. Legend: Circles, squares and triangles indicate pregnant women in their first, second and third trimesters, respectively. Diamond markers indicate lactating women. Dashed horizontal lines denote cut offs for elevated CRP (first trimester and lactation = 28.5 nmol/L; second trimester = 193.3 nmol/L; third trimester = 77.1 nmol/L) [20]

Mean CRP concentration was higher in pregnancy than lactation (Table 1), but did not differ between first (35.1 ± 8.4 nmol/L; median 31.4; interquartile range (IQR) 51.4), second (58.4 ± 9.7 nmol/L; median 32.4; IQR 57.1) and third (50.0 ± 5.7 nmol/L; median 37.6; IQR 62.8) trimesters. Based on trimester-specific cut-offs, elevated CRP occurred in 63.6%, 7.0% and 22.7% of first, second and third trimester women, respectively, and in 30% of lactating mothers (Fig. 2).

CRP did not differ among women according to age, parity or BMI classifications. CRP concentrations were compared between binary categories for a variety of maternal variables (Table 2). In pregnancy, CRP concentrations were higher in women with caries (Table 2) and lower in those with *Ascaris* (Table 2). During lactation, CRP concentrations were higher in women with above normal vitamin A (>1.5 μmol/L) and lower in grand-multiparous women and those with caries (Table 2).

**Table 2:** CRP concentrations (nmol/L) in the presence and absence of maternal conditions in pregnancy and lactation^1^

	PREGNANCY		LACTATION	
Condition	Yes	No	Yes	No
Environmental hazards				
Wood smoke	52.2 ± 4.9 (*n* = 114)	41.7 ± 19.8 (*n* = 6)	28.0 ± 3.7 (*n* = 93)	24.4 ± 8.4 (*n* = 6)
Fieldwork	52.3 ± 7.5 (*n* = 60)	51.0 ± 5.9 (*n* = 60)	24.0 ± 4.7 (*n* = 41)	30.4 ± 5.0 (*n* = 58)
Micronutrient status				
Folic acid < 10 nmol/L	61.1 ± 11.6 (*n* = 25)	49.2 ± 5.1 (*n* = 95)	30.1 ± 6.7 (*n* = 31)	26.7 ± 4.2 (*n* = 68)
Vitamin B_12_ < 150 pmol/L	48.8 ± 4.9 (*n* = 103)	68.7 ± 15.5 (*n* = 17)	24.6 ± 4.5 (*n* = 46)	30.5 ± 5.4 (*n* = 53)
Vitamin A < 1.05 μmol/L	59.5 ± 8.9 (*n* = 46)	47.1 ± 5.3 (*n* = 73)	30.2 ± 11.7 (*n* = 18)	27.3 ± 3.6 (*n* = 79)
Vitamin A > 1.5 μmol/L	35.6 ± 6.5 (*n* = 22)	55.2 ± 5.5 (*n* = 98)	30.6 ± 5.5^a^ (*n* = 39)	26.0 ± 4.8^b^ (*n* = 58)
Vitamin D < 50 nmol/L	54.2 ± 5.8 (*n* = 82)	46.1 ± 8.2 (*n* = 38)	25.7 ± 3.9 (*n* = 68)	32.3 ± 7.5 (*n* = 31)
Clinically detected Infections				
Caries	74.0 ± 12.2^a^ (*n* = 25)	45.8 ± 4.9^b^ (*n* = 95)	21.4 ± 8.3^b^ (*n* = 18)	29.2 ± 3.9^a^ (*n* = 81)
Scabies	53.1 ± 11.8 (*n* = 26)	51.2 ± 5.1 (*n* = 94)	25.8 ± 9.5 (*n* = 8)	27.9 ± 3.8 (*n* = 91)
Laboratory detected infections				
AB/UTI	53.5 ± 7.1 (*n* = 63)	47.1 ± 6.1 (*n* = 55)	26.0 ± 6.4 (*n* = 34)	30.0 ± 4.5 (*n* = 60)
Bacterial vaginosis	52.5 ± 6.0 (*n* = 73)	50.8 + 7.9 (*n* = 46)	21.7 ± 4.3 (*n* = 50)	22.8 ± 5.6 (*n* = 29)
*Lactobacillus*	48.8 ± 6.7 (*n* = 59)	54.8 ± 6.8 (*n* = 60)	22.2 ± 6.9 (*n* = 21)	22.0 ± 3.9 (*n* = 58)
*Bacteroides*/*Gardnerella*	51.5 ± 4.8 (*n* = 114)	59.4 ± 29.6 (*n* = 5)	22.5 ± 3.5 (*n* = 77)	5.2 ± 4.3 (*n* = 2)
*Mobiluncus*	48.8 ± 4.9 (*n* = 95)	63.9 ± 13.7 (*n* = 24)	20.6 ± 3.4 (*n* = 69)	31.4 ± 13.4 (*n* = 10)
Vaginal Trichomoniasis	52.8 ± 5.8 (*n* = 88)	49.1 ± 8.0 (*n* = 31)	23.0 ± 3.7 (*n* = 72)	12.0 ± 3.7 (*n* = 7)
Vaginal yeast infection	47.2 ± 10.3 (*n* = 33)	53.3 ± 5.2 (*n* = 87)	16.0 ± 5.7 (*n* = 9)	22.9 ± 3.8 (*n* = 70)
Vaginal Diplococcal infection	64.4 ± 14.3 (*n* = 24)	48.6 ± 4.7 (*n* = 95)	18.1 ± 4.5 (*n* = 25)	23.9 ± 4.5 (*n* = 54)
*Ascaris*	44.0 ± 8.1^b^ (*n* = 39)	55.3 ± 5.8^a^ (*n* = 81)	35.0 ± 11.9 (*n* = 4)	35.9 ± 10.4 (*n* = 19)
Hookworm	55.9 ± 6.5 (*n* = 68)	46.1 ± 6.8 (*n* = 52)	34.8 ± 14.7 (*n* = 11)	36.5 ± 10.6 (*n* = 12)
*Trichuris*	33.3 ± 10.9 (*n* = 15)	54.3 ± 5.1 (*n* = 105)	49.4 ± 27.3 (*n* = 2)	34.4 ± 9.4 (*n* = 21)

^1^Values are means ± *SE*; different letter superscripts are significantly different at *P* < 0.05

In pregnancy, CRP was positively correlated with NLR (*r*_s_ = 0.24, *P* = 0.008), platelet count (*r*_s_ = 0.20, *P* = 0.032), plateletcrit (*r*_s_ = 0.23, *P* = 0.012), IL6 (*r*_s_ = 0.23, *P* = 0.011), IL10 (*r*_s_ = 0.25, *P* = 0.005), IL12 (*r*_s_ = 0.28, *P* = 0.002), IL13 (*r*_s_ = 0.25, P = 0.005) and TNFα (*r*_s_ = 0.22, *P* = 0.015). In lactation, CRP was positively correlated with numbers of neutrophils (*r*_s_ = 0.30, *P* = 0.002), NLR (*r*_s_ = 0.29, *P* = 0.003), platelet count (*r*_s_ = 0.40, *P* < 0.0001), plateletcrit (*r*_s_ = 0.34, *P* = 0.0006) and IL4 (*r*_s_ = 0.20, *P* = 0.042), and negatively correlated with number of eosinophils (*r*_s_ = −0.25, *P* = 0.010) and MPV (*r*_s_ = −0.27, *P* = 0.006).

To determine whether CRP was associated with other markers of inflammation and to confirm significant correlations, multiple regression analyses controlling for gestational age, parity and BMI during pregnancy revealed a positive association of log CRP with NLR and plateletcrit and accounted for 14.5% of the variability in CRP (Table 3). During lactation, log CRP was also positively associated with plateletcrit and negatively associated with number of eosinophils when controlling for weeks postpartum, parity and BMI and the model explained 27.5% of variability in CRP. No cytokines emerged in either model (Table 3).

**Table 3:** Multiple regression of Log CRP with indicators of inflammation in pregnant and lactating women

CRP (nmol/L) in pregnant women^1^	Coefficient ± *SE*	*P*	Overall Model
BMI, kg/m^2^	−0.06 ± 0.04	0.039	*n* = 114 F_5, 108_ = 4.85 *P* = 0.0005 Adj. R^2^ = 0.145 VIF = 1.18
Neutrophil/lymphocyte ratio	0.14 ± 0.07	0.041	
Plateletcrit, %	0.06 ± 0.02	0.024	
IL6, pg/mL	0.01 ± 0.009	0.122	
IL13, pg/mL	0.02 ± 0.01	0.142	
Constant	2.83 ± 0.94	0.003	
CRP (nmol/L) in lactating women^2^	Coefficient ± *SE*	*P*	Overall Model
Parity	−0.12 ± 0.05	0.026	*n* = 97 F_6, 90_ = 7.09 *P* < 0.0001 Adj. R^2^ = 0.275 VIF = 1.12
BMI, kg/m^2^	0.06 ± 0.04	0.960	
Neutrophil/lymphocyte ratio	0.29 ± 0.16	0.072	
Eosinophils × 10^3^/mm^3^	−0.61 ± 0.19	0.002	
Plateletcrit, %	0.04 ± 0.02	0.016	
IL4	0.03 ± 0.02	0.144	
Constant	−0.29 ± 1.23	0.813	

^1^Variables that were explored but did not enter the pregnancy model (*P* > 0.15): Gestational age, parity, IL4, IL10, IL12, IL17, TNFα

^2^Variables that were explored but did not enter the lactation model (*P* > 0.15): Weeks postpartum, IL17

### Role of maternal factors, infections and vitamin deficiencies as determinants of CRP

During pregnancy, our final multiple linear regression model explained 18.9% of variation in log CRP (Table 4). Wood smoke, presence of caries and hookworm were positively associated with CRP; presence of *Ascaris*, and scores for vaginal *Lactobacillus* and *Bacteroides/Gardnerella* were negatively associated with CRP. Using trimester specific cut-offs for elevated CRP (Table 4), our multiple logistic regression model showed that presence of caries, higher diplococcal infection score and greater indoor wood smoke exposure were associated with higher likelihood of elevated CRP, and higher gestational age was associated with a lower likelihood of elevated CRP. The Pseudo R^2^ was 0.243.

**Table 4:** Multiple linear and logistic regression models for Log CRP and elevated CRP in pregnant women

CRP (nmol/L) in pregnant women^1^	Coefficient ± *SE*	*P*	95% *CI*	Overall Model
Wood smoke, h/d	0.15 ± 0.07	0.032	0.01, 0.30	*n* = 116 F_9, 106_ = 3.99 *P* = 0.0002 Adj. R^2^ = 0.189 VIF = 1.52
Caries, presence	0.66 ± 0.26	0.012	0.15, 1.12	
*Lactobacillus,* score	−0.27 ± 0.11	0.020	−0.5, −0.04	
*Bacteroides/Gardnerella,* score	−0.35 ± 0.14	0.019	−0.64, −0.06	
*Mobiluncus,* score	0.20 ± 0.10	0.059	−0.008, 0.42	
*Ascaris*, presence	−0.73 ± 0.23	0.002	−1.20, −0.27	
Hookworm, presence	0.56 ± 0.22	0.014	0.11, 1.02	
*Trichuris*, presence	−0.57 ± 0.33	0.092	−1.24, 0.09	
Vitamin A < 1.05 μmol/L	0.34 ± 0.22	0.122	−0.09, 0.78	
Constant	1.55 ± 0.06	0.019	0.26, 2.85	
Elevated CRP in pregnant women^2^	*OR* ± *SE*	*P*	95% *CI*	Overall model
Gestational age	0.92 ± 0.03	0.022	0.87, 0.98	*n* = 116 *P* = 0.0006 Pseudo R^2^ = 0.243 VIF = 1.09
Wood smoke, h/d	1.50 ± 0.29	0.034	1.03, 2.19	
Caries, presence	3.86 ± 2.46	0.034	1.10, 13.4	
Vaginal yeast, score	0.36 ± 0.18	0.050	0.13, 0.99	
Diplococcal infection, score	2.04 ± 0.53	0.007	1.22, 3.42	
Folic acid < 10 nmol/L	3.53 ± 2.34	0.057	0.96, 12.9	
Vitamin A < 1.05 μmol/L	2.45 ± 1.43	0.124	0.78, 7.72	
Vitamin D < 50 nmol/L	2.74 ± 1.91	0.148	0.69, 10.70	

^1^Variables that were explored but did not enter the linear regression model for CRP (nmol/L) in pregnancy (*P* > 0.15): Gestational age, parity, BMI (kg/m^2^), fieldwork (h/d), presence of scabies and AB/UTI, score of trichomoniasis, vaginal yeast and diplococcal infection, folic acid < 10 nmol/L, vitamin B_12_ < 150 pmol/L, vitamin A > 1.5 μmol/L. Vitamin D < 50 nmol/L

^2^Variables that were explored but did not enter the logistic regression model for elevated CRP (>28.50 nmol/L, > 193.34 nmol/L and > 77.14 nmol/L in the first, second and third trimesters, respectively [20]) in pregnancy (*P* > 0.15): Parity, BMI (kg/m^2^), fieldwork (h/d), presence of scabies, AB/UTI, *Ascaris*, hookworm, *Trichuris*, score of *Lactobacillus*, *Bacteroides/Gardnerella*, *Mobiluncus*, trichomoniasis, vitamin B_12_ < 150 pmol/L, vitamin A > 44.3 μmol/L

During lactation, our composite multiple linear regression model captured 24.4% of the variability in log CRP. Folic acid deficiency was positively associated with CRP; the number of eosinophils, the *Mobiluncus* score and parity were negatively associated with CRP (Table 5). Our multiple logistic regression model for elevated CRP in lactation had a Pseudo R^2^ of 0.243 (Table 5). Higher *T. vaginalis* score and BMI were associated with a higher likelihood of elevated CRP, and a higher number of eosinophils and parity were associated with lower likelihood of elevated CRP.

**Table 5:** Multiple linear and logistic regression models for Log CRP and elevated CRP in lactating women

CRP (nmol/L) in lactating women^1^	Coefficient ± *SE*	*P*	95% *CI*	Overall model
Parity	−0.17 ± 0.06	0.006	−0.29, −0.05	*n* = 78 F_8, 69_ = 4.11 *P* = 0.0005 Adj. R^2^ = 0.244 VIF = 1.18
Fieldwork, h/d	0.08 ± 0.05	0.119	−0.02, 0.19	
Eosinophils, number	−0.48 ± 0.20	0.019	−0.88, −0.08	
Caries, presence	−0.61 ± 0.34	0.080	−1.30, 0.07	
*Mobiluncus*, score	−0.31 ± 0.13	0.018	−0.57, −0.05	
Folic acid < 10.0 nmol/L	0.64 ± 0.29	0.033	0.05, 1.23	
Vitamin A < 1.05 μmol/L	−0.73 ± 0.38	0.061	−1.50, 0.03	
Vitamin A > 1.50 μmol/L	0.54 ± 0.31	0.085	−0.07, 1.16	
Constant	3.57 ± 0.50	<0.0001	2.56, 4.57	
Elevated CRP in lactating women^2^	*OR* ± *SE*	*P*	95% *CI*	Overall model
Parity	0.53 ± 0.12	0.007	0.34, 0.84	*n* = 78 *P* = 0.001 Pseudo R^2^ = 0.243 VIF = 1.08
BMI	1.31 ± 0.16	0.029	1.02, 1.66	
*Mobiluncus,* score	0.51 ± 0.18	0.066	0.25, 1.04	
Eosinophils, number	0.24 ± 0.16	0.031	0.06, 0.88	
Trichomoniasis, score	2.52 ± 0.97	0.016	1.18, 5.36	

^1^Variables that were explored but did not enter the linear regression model for CRP (nmol/L) in lactation (*P* > 0.15): Weeks post-partum, BMI (kg/m^2^), wood smoke (h/d), presence of caries, score of vaginal *Lactobacillus*, *Bactaroides/Gardnerella*, *T.vaginalis*, yeast and diplococcal infection, vitamin B_12_ < 150 pmol/L, vitamin D < 50 nmol/L

^2^Variables that were explored but did not enter the logistic regression model for elevated CRP in lactation (*P* > 0.15): Weeks post-partum, wood smoke exposure (h/d), fieldwork (h/d) presence of caries, score of vaginal *Lactobacillus*, *Bacteroides/Gardnerella*, *Mobiluncus*, yeast and diplcoccal infection, folic acid < 10 nmol/L, vitamin B_12_ < 150 pmol/L, vitamin A < 1.05 μmol/L, vitamin A > 1.5 μmol/L, vitamin D < 50 nmol/L

## Discussion

Several key findings emerged from this study. First, our results differentiated between those infections that were associated with higher CRP and those associated with lower CRP. During pregnancy, dental caries, hookworm and vaginal diplococcal infection were associated with higher CRP or increased the odds of trimester-specific elevated CRP whereas *Ascaris*, *Lactobacillus* and *Bacteroides/Gardnerella* were negatively associated with an elevated CRP. During lactation, vaginal trichomoniasis was associated with an elevated CRP whereas vaginal *Mobiluncus* and eosinophil counts were associated with lower CRP and/or lower odds of an elevated CRP. Second, although deficiencies of vitamins A, B_12_, D and folic acid were common, only folic acid deficiency was associated with higher CRP in lactating women. Third, higher daily indoor wood smoke exposure was positively associated with CRP and increased the odds of elevated CRP in pregnancy. Together, these results show that, after correcting for the normal elevation of CRP during pregnancy, the systemic CRP concentration is influenced by the mix of diverse infections, folic acid deficiency and maternal wood smoke exposure in this vulnerable population.

Our finding that CRP was positively associated with NLR and plateletcrit is consistent with their usefulness as markers of inflammation [2, 52]. NLR is thought to be an early marker of bacteremia given its positive association with CRP [53], and NLR has also been associated with placental inflammation when NLR ≥ 6.48 and CRP ≥ 71 nmol/L [54]. Activation of platelets occurs in response to inflammatory stimuli [55], and activated platelets are enlarged resulting in higher plateletcrit [56]. Our negative association of eosinophils with CRP during lactation is consistent with the production of Th2 cytokines, particularly IL4, by eosinophils [57]; both Th2 and IL4 are required for protection against intestinal nematodes and for suppression of a Th1 pro-inflammatory cytokine response [58].

Although CRP was positively correlated with four cytokines during pregnancy and three during lactation, cytokines did not emerge in the multiple regression model. We interpret this to mean that CRP was a more specific marker of inflammation than cytokines in this population. Thus, we suggest that plateletcrit and NLR that have been useful for the diagnosis of obstetric pathologies in tertiary care [39, 59] would be alternatives to evaluate inflammation in pregnant and lactating women in settings where a complete blood count, but not CRP measurement, is possible.

Among the four vitamin deficiencies detected in this population, only folic acid deficiency was associated with higher CRP and only in lactating women. This is consistent with the observation that folate intake was protective against elevation of CRP above 28.5 nmol/L in lactating women from Kenya [60] and that young Vietnamese women with higher folic acid intake had lower CRP [61]. The absence of a relationship between folic acid deficiency and CRP during pregnancy is most likely because women in our study were receiving folic acid supplementation during pregnancy. These findings suggest that there may be a need to extend folate supplementation beyond the 3 months post-partum established in the national protocol.

Among the many infections detected [42], several were associated with higher CRP and/or a higher likelihood of an elevated CRP. In pregnancy, the finding that caries was associated with higher CRP is consistent with reports showing that periodontal disease is associated with elevated CRP in pregnancy [62] and correlated with CRP [63]. Furthermore, aggressive vaginal microorganisms such as *Diplococcus* and *T. vaginalis* were found to induce a systemic inflammatory response. To our knowledge this is the first report of diplococcal infection being associated with higher CRP in pregnancy. Also, the positive association detected between trichomoniasis and CRP in lactation extends the findings of a systemic inflammatory response measured through CRP and granulocyte-macrophage colony-stimulating factor found in pregnant women with vaginal trichomoniasis [64]. It is also consistent with a mouse study that demonstrated signs of systemic inflammation associated with trichomoniasis [65].

One striking observation was that several vaginal bacteria were associated with lower, not higher CRP. The negative relationship between *Lactobacillus* and CRP in pregnancy is consistent with the observation that *Lactobacillus* dominates the vaginal flora in healthy women [66] and that many species of *Lactobacillus* are considered to be protective [67]. It is also consistent with the lower amount of *Lactobacillus* but higher CRP in pregnant compared with non-pregnant women in the US [68]. In addition, however, the typical pathogenic bacteria associated with BV, *Bacterioides/Gardnerella* and *Mobiluncus* [69], were also associated with lower CRP in pregnancy and lactation, respectively. As BV has been linked with inflammation during pregnancy [70, 71], we would have expected both *Bacteriodes/Gardnerella* and *Mobiluncus* to be associated with higher CRP but they were not. Together, these contrasting findings show that the vaginal tract has organisms that both increase and decrease CRP and is supported by a recent study showing women with BV and adverse pregnancy outcomes had different vaginal microbiota profiles compared to women with BV and no adverse pregnancy outcomes [71], indicating different host-parasite adaptations depending the type of vaginal microorganisms. This requires further investigation.

We were also intrigued that *Ascaris* was negatively associated with CRP whereas hookworm was positively associated with CRP in pregnancy. There is considerable evidence of the anti-inflammatory effect of *Ascaris* based on the in vitro suppressive effects of *Ascaris suum*-derived protein (PAS-1) on pro-inflammatory cytokine production [72], the down-regulation by low intensity *Ascaris* infection of the IL6 response to intestinal giardiasis [73], the anti-inflammatory effect of *Ascaris* antigen on cytokine production by peripheral mononuclear cells from children when co-incubated with allergens [74], and the consistent protective effect of *Ascaris* against allergic sensitization found in a meta-analysis [75]. However, to our knowledge, this is the first report of a negative association of CRP with *Ascaris* during pregnancy. Also, the possibility that this anti-inflammatory influence would reduce CRP in the presence of multiple infections and vitamin deficiencies has not been previously considered. This may have important public health implications during pregnancy given the health risks of pathologies often associated with inflammation.

There are two reasons why we would have also expected a negative association of hookworm with CRP. First, secretory products released by adult hookworms during feeding can down-regulate the inflammatory response in humans by suppressing TNFα secretion [76]. Second, although experimental hookworm infection in a human volunteer showed a transient increase in CRP as well as pro-inflammatory (IFNγ) and T helper 2 (Th2) cytokines (IL5, IL13) soon after infection, this response was dampened once adult worms had reached the intestine and started shedding eggs [77]. It is unlikely that hookworm was a transient infection in women in our study, as the prevalence based on egg counts was over 50%, indicating ongoing transmission [42]. The observed positive association between CRP and presence of hookworm in pregnancy may be explained by different degrees of tissue damage caused by the adult worms. In contrast to the non-invasive manner by which adult *Ascaris* worms feed [78], hookworm adults invade the mucosa and submucosa of the small intestine where the release of hydrolases and mechanical injury associated with feeding results in considerable tissue damage [79]. An additional justification for the different response of CRP between hookworm and *Ascaris* in pregnancy is explained by the capacity of the adult hookworm *Necator americanus* to produce proteases that specifically cleave the eosinophil chemoattractant eotaxin [80] whereas *Ascaris* induces higher transcription levels of eotaxin at least in pigs [81].

In this population where wood is used as fuel for cooking, increased hours of wood smoke exposure during pregnancy increased the odds of elevated CRP. Studies from India have reported that wood fuel use is associated with low birth weight [82], more frequent stillbirth and increased risk of preterm delivery [83], outcomes that are in turn associated with elevated CRP [84, 85]. To our knowledge, this is the first report of an association between biofuel exposure and inflammation during pregnancy, although the finding is consistent with a study of women living in rural India that found increased serum CRP concentration in those exposed to wood smoke [12].

## Strengths and limitations

This cross sectional screening of indigenous pregnant and lactating women in a remote outpatient setting allowed us to collect data on biomarkers of inflammation, infection and nutritional status and to associate these with CRP concentration during the three trimesters of pregnancy and 6 months postpartum. Nevertheless, we acknowledge the following limitations. The cross-sectional design precluded us from monitoring CRP over time and from relating CRP to pregnancy outcomes. The results of our multiple logistic regression models for trimester-specific elevations in CRP in pregnancy may need to be viewed with caution because of the lack of a consensus on trimester-specific cut-offs for CRP [85]. We were unable to explicitly consider intestinal nematodes in our regression models during lactation due to the high proportion of women who did not provide a faecal sample.

## Conclusions

Both within the intestine and within the vaginal tract, some organisms raised and others lowered systemic CRP. This highlights the complexity of the associations of pathogens and CRP, and raises questions about how to interpret CRP in settings with multiple infections. It is possible that treatment of ascariasis may inadvertently increase CRP and that the treatment of BV may lead to the opportunistic growth of concurrent/more aggressive microorganisms and to an elevation in CRP, which could adversely affect pregnancy outcomes. Further, folic acid deficiency may play an under recognized role in leading to increased CRP concentrations in lactating women, in which case it might be recommended that folic acid supplementation be extended beyond 3 mo postpartum. Finally, consideration should be given to reducing exposure to indoor wood smoke particularly during pregnancy, given its role in increasing CRP.

## Additional file

Additional file 1: Multilingual abstracts in the six official working languages of the United Nations. (PDF 829 kb)

## Abbreviations

AB/UTI: Asymptomatic bacteriuria/urinary tract infection
BMI: Body mass index
BV: Bacterial vaginosis
CRP: C-reactive protein
HIV: Human immunodeficiency virus
IL: Interleukin
INDICASAT: Institute of Scientific Research and High Technology Services (Panama)
IQR: Interquartile range
MPV: Mean platelet volume
NLR: Neutrophil/Lymphocyte ratio
PDW: Platelet distribution width
SENACYT: Secretaría Nacional de Ciencia, Tecnología e Innovación
Th2: Lymphocyte T helpers 2
VIF: Variance inflation factor
WBC: White blood cells
WHO: World Health Organization
