# Supplementary material for: C-reactive protein is differentially modulated by co-existing infections, vitamin deficiencies and maternal factors in pregnant and lactating indigenous Panamanian women
Source: Infect Dis Poverty. 2017 Jun 2;6:94. doi: 10.1186/s40249-017-0307-1 (PMC5455098; doi:10.1186/s40249-017-0307-1)
Supplement: Additional file 1: — Multilingual abstracts in the six official working languages of the United Nations. (PDF 829 kb) [file 40249_2017_307_MOESM1_ESM.pdf]

Translation of the abstract into the five official working languages of the United Nations

تمايز تضمين البروتين الإرتكاسي سي عن طريق العدوى المتعايشة، ونقص الفيتامينات والعوامل المؤثرة التي لها علاقة بالأم في النساء البنميات من سكان البلاد الأصليين الحوامل والمرضعات

دوريس جونزاليس فيرنانديز، إميريتا ديل كارمن بونس، دلفينا رويدا، أوداليس تيريزا سينيستيرا، انريكي موريللو، مارلين إ. سكوت[6] وكريستين ج. كوسكي

ملخص

خلفية: لا تتضح فائدة البروتين الإرتكاسي سي كعلامة غير محددة للالتهاب خلال فترة الحمل والرضاعة في السكان المحرومين حيث تشيع العدوى المتزامنة مع الإصابة بنقص الفيتامينات.

الطريقة: هذه دراسة شاملة لمختلف القطاعات والفئات في بنما شملت 120 سيدة حامل و99 سيدة مرضعة من دائرة نجابي-بوجلي من 14 منطقة ريفية في بنما. تم قياس تاريخ الولادة، التعرض لدخان الخشب في الأماكن المغلقة، العمل الميداني، مؤشر كتلة الجسم، فيتامينات أ، ب 12، د، حمض الفوليك، علامات الالتهاب (البروتين الإرتكاسي سي، العدلات / نسبة الخلايا اللمفاوية (NLR)، الصفائح الدموية الحرجة والسيتوكينات). أظهرت الانحدارات المتعددة اقتران البروتين الإرتكاسي سي مع علامات الالتهاب وبالإضافة إلى اقتران البروتين الإرتكاسي سي مع زيادة بروتين الإرتكاسي سي وفقا لانخفاضات مرتبطة بأُثْلوث الحمل مع العوامل المرتبطة بالأم، والعدوى ونقص الفيتامينات.

النتائج: كان البروتين الإرتكاسي سي أعلى في الحمل (51.4 ± 4.7 نانومول / لتر) عن الرضاعة (27.8 ± 3.5 نانومول / لتر) وارتفع عن الثلث الانخفاضات المرتبطة بأُثْلوث الحمل في 21٪ من الحوامل و30٪ من النساء المرضعات. شاع نقص الفيتامينات (فيتامين أ 29.6٪، فيتامين د 68.5٪، فيتامين ب 12 68٪، حمض الفوليك 25.5٪)، وكان أكثر من 50٪ من النساء يعانين من اثنين أو أكثر من أوجه القصور المتزامنة وكذلك إصابات متعددة. أبرزت نماذج الانحدار المتعدد الاختلافات في المتغيرات المرتبطة بالبروتين الإرتكاسي سي بين الحمل والرضاعة. في الحمل، ارتبط البروتين الإرتكاسي سي إيجابيا مع زيادة التعرض لدخان الخشب داخل المنزل، والتسوس ودودة الأنكلستوما وارتبط سلبيا مع كمية الاسكاريس والعصية اللبنية الحمضية للمهبل والعصوانية / الغاردنريلة. واتساقا مع ذلك، فزيادة التعرض لدخان الخشب، والتسوس، وكذلك ارتفاع درجة الإصابة بالمكورة المزدوجة زادت احتمالات كون الأُثْلوث به ارتفاع في تركيزات البروتين الإرتكاسي سي، في حين زيادة عمر الحمل زاد احتمال كون الأُثْلوث منخفض في نسبة البروتين الإرتكاسي سي. أثناء الرضاعة، يرتبط نقص حمض الفوليك مع ارتفاع البروتين الإرتكاسي سي أثناء التعادل، ارتبط عدد الكريات البيضاء الحمِضة ونسبة بكتيريا مبلونكوس بانخفاض البروتين الإرتكاسي سي. أيضا، زاد ارتفاع مؤشر كتلة الجسم والمشعرة المهبلية درجة من احتمال ارتفاع البروتين الإرتكاسي سي في حين ارتبط ارتفاع التكافؤ وعدد الكريات البيضاء الحمِضة مع قلة احتمال ارتفاع البروتين الإرتكاسي سي.

الاستنتاجات: تعمل العدوى زيادة وانخفاض تركيزات البروتين الإرتكاسي سي على حد سواء في الأمهات الحوامل والمرضعات. ارتبط نقص حمض الفوليك خلال فترة الرضاعة فقط مع ارتفاع تركيزات البروتين الإرتكاسي سي. ويتوجب الحذر عند تفسير تركيزات البروتين الإرتكاسي سي في النساء الحوامل والمرضعات الذين يعانين من نقص المواد الغذائية متلازمنا مع إصابات متعددة.

Translated from English version into Arabic by Mahmoud Sami, through

TRANSLATORS
WITHOUT BORDERS

在妊娠期和哺乳期巴拿马原住妇女中 **C-**反应蛋白被合并感染、维生素缺乏和孕期因素调控

Doris González-Fernández, Emérita del Carmen Pons, Delfina Rueda, Odalis Teresa Sinisterra,

Enrique Murillo, Marilyn E. Scott6 and Kristine G. Koski

摘要

引言：在合并感染和维生素缺乏的贫困人群中，C-反应蛋白作为妊娠和哺乳期非特异性的炎症标志物的有效性是不确定的。

方法：该横断面研究募集了来自巴拿马农村的 14 个社区的 120 名妊娠期妇女和 99 名哺乳期 Ngäbe-Buglé 妇女。我们对孕产史、室内炊烟暴露情况、野外工作情况、体重指数、维生素 A、、B12、D，叶酸以及炎症标志物（C-反应蛋白、中性粒细胞/淋巴细胞比例、血小板压积以及细胞因子）进行了评估测定。多重回归分析探究了 C-反应蛋白与其他炎症标志物的关联以及基于妊娠三期特定的界限，C-反应蛋白的升高与孕期因素，感染和维生素缺乏之间的关联。

结果：妊娠期女性(51.4±4.7 nmol/L)的 C-反应蛋白高于哺乳期女性(27.8±3.5 nmol/L)，并且 21%妊娠期妇女和 30%哺乳期妇女的 C-反应蛋白高于妊娠期界限。维生素缺乏十分常见，缺乏维生素 A、D 和叶酸的分别占 29.6%、68.5%和 25.5%，并且超过 50%的女性有两种或同时存在多种缺乏及多种感染。多重回归模型显示，与 C-反应蛋白相关的变量在妊娠期和哺乳期存在差异。在妊娠期，C-反应蛋白量与室内炊烟暴露、龋齿以及钩虫成正相关，与蛔虫、阴道乳酸杆菌（*Lactobacillus*）、拟杆菌（*Bacteroides*）/加德纳菌（*Gardnerella*）评分成负相关。与此相一致，更多的炊烟暴露、龋齿和较高的双球菌感染评分增加了妊娠期高 C-反应蛋白浓度的发生率，然而更长的孕龄降低了妊娠期高 C-反应蛋白的发生率。在哺乳期，叶酸缺乏与较高的 C-反应蛋白相关。然而，分娩次数、嗜酸粒细胞数量和动弯杆菌（*Mobiluncus*）评分与更低的 C-反应蛋白相关。同样，较高的体重指数和阴道毛滴虫评分增加了 C-反应蛋白升高的发生率，而较多的分娩次数和中性粒细胞数目降低了 C-反应蛋白升高的发生率。

结论：对于妊娠和哺乳期母亲，感染既可增加也可降低 C-反应蛋白浓度。在哺乳期，仅叶酸缺乏与较高的 C-反应蛋白浓度相关。对于合并营养缺乏和多重感染的妊娠期和哺乳期妇女，应当慎重解释 C-反应蛋白浓度。

Translated from English version into Chinese by Lei Sun, edited by Yang Pin, through

CHINESE CENTER FOR DISEASE CONTROL AND PREVENTION
NATIONAL INSTITUTE OF PARASITIC DISEASES

La protéine C-réactive est modulée de façon différentielle par les infections coexistantes, les carences en vitamines et les facteurs maternels chez les femmes panaméennes autochtones enceintes et allaitantes

Doris González-Fernández, Emérita del Carmen Pons, Delfina Rueda, Odalis Teresa Sinisterra, Enrique Murillo, Marilyn E. Scott[6] and Kristine G. Koski

Résumé

Contexte: L'utilité de la protéine C-réactive (PCR) comme marqueur non spécifique d'inflammation au cours de la grossesse et l'allaitement n'apparaît pas clairement dans les populations défavorisées, où les infections coexistantes et les carences en vitamines sont courantes.

Méthodes: Cette étude transversale effectuée à Panama a recruté 120 femmes enceintes et 99 femmes allaitantes Ngäbe-Buglé de 14 communautés des zones rurales du Panama. On a évalué leur historique obstétrical, leur exposition à la fumée de bois à l'intérieur des habitations, leur travail sur le terrain, leur IMC, les vitamines A, B_{12}, D, ainsi que l'acide folique et les marqueurs d'inflammation (PCR, taux de neutrophiles/lymphocytes [TNL], les indices plaquettaires et les cytokines). Les régressions multiples ont exploré les associations de PCR avec d'autres marqueurs inflammatoires et les associations de PCR et de PCR élevé basé sur des seuils trimestriels spécifiques avec les facteurs maternels, les infections et les carences en vitamines.
Résultats: La PCR était plus élevée pour la grossesse (51,4 ± 4,7 nmol/L) que pour l'allaitement (27,8 ± 3,5 nmol/L) et s'élevait au-dessus des seuils trimestriels spécifiques chez 21% des femmes enceintes et 30% des femmes allaitantes. Les carences en vitamines étaient courantes (vitamine A 29,6%; vitamine D 68,5%; vitamine B_{12} 68%; acide folique 25,5%) et plus de 50% des femmes avaient deux ou plus de deux carences concomitantes ainsi que de multiples infections. Les modèles de régression multiple ont mis en évidence des différences de variables, associées à la PCR, entre la grossesse et la lactation. Pendant la grossesse, la PCR était associée positivement à une grande exposition à la fumée de bois à l'intérieur des habitations, aux caries et à l'ankylostomiase, et associée négativement à l'*Ascaris* et au *Lactobacillus* vaginal, et aux résultats de *Bacteroides/Gardnerella*. En conformité avec ceci, une plus grande exposition à la fumée de bois, des caries ainsi que des résultats d'infection diplococcale ont augmenté les chances de concentrations trimestrielles élevées de PCR, alors que l'âge gestationnel a abaissé la probabilité de PCR trimestrielle élevée. Au cours de la lactation, la carence en acide folique a été associée à une PCR plus élevée, alors que la parité, le nombre d'éosinophiles et le résultat du *Mobiluncus* ont été associés à une PCR plus basse. Egalement, un IMC plus élevé et des résultats de *Trichomonas vaginalis* ont augmenté la probabilité élevée de PCR, alors qu'une parité et un nombre d'éosinophiles plus élevé ont été associés à une probabilité plus basse de PCR élevée.
Conclusions: Les infections élèvent et abaissent les concentrations de PCR chez les femmes enceintes et allaitantes. Seule une carence en acide folique au cours de la lactation a été associée à des concentrations plus élevées de PCR. Il faut interpréter avec prudence les concentrations de PCR chez les femmes enceintes et allaitantes qui ont des carences nutritionnelles coexistantes et de multiples infections.

Translated from English version into French by Ode Laforge, through

TRANSLATORS
WITHOUT BORDERS

Избирательное влияние на С-реактивный белок сосуществующих инфекций, авитаминоза и материнских факторов у беременных и кормящих коренных женщин Панамы

Дорис Гонзалез-Фернандез (Doris González-Fernández), Эмерита дель-Кармен Понс (Emérita del Carmen Pons), Дельфина Руеда (Delfina Rueda), Одалис Тереса Синистерра (Odalis Teresa Sinisterra), Энрике Мурильо (Enrique Murillo), Мэрилин Е. Скотт (Marilyn E. Scott)[6] и Кристин Дж. Коски (Kristine G. Koski)

Аннотация

Краткое описание. Значимость С-реактивного белка (CRP) в качестве неспецифического показателя воспаления во время беременности и кормления не установлена для бедных слоев населения, где одновременно распространены инфекции и авитаминоз.

Методы. Для этого перекрёстного исследования были набраны 120 беременных и 99 кормящих женщин народности нгабе-бугле (Ngäbe-Buglé) из 14 общин в сельской местности Панамы. Были оценены акушерский анамнез, вдыхание древесного дыма в помещении, работа в поле, индекс массы тела (ИМТ), витамины A, B_{12}, D, фолиевая кислота и показатели воспаления (CRP, индекс соотношения нейтрофилов и лимфоцитов, тромбокрит и цитокины). С помощью множественной регрессии были изучены связь CRP с другими показателями воспаления и зависимость повышенного CRP от материнских факторов, инфекции и авитаминоза для каждого триместра.

Результаты. CRP был выше во время беременности (51,4 ± 4,7 пмоль/л), чем при кормлении (27,8 ± 3,5 пмоль/л), и превышал нормы для каждого триместра у 21% беременных и 30% кормящих женщин. Распространён авитаминоз (витамин A 29,6%; витамин D 68,5%; витамин B_{12} 68%; фолиевая кислота 25,5%). Свыше 50% женщин имели два или более вида витаминной недостаточности одновременно, а также различные инфекции. Модели множественной регрессии продемонстрировали разницу в связанных с CRP показателях между беременностью и кормлением. При беременности CRP был положительно связан с более продолжительным вдыханием древесного дыма в помещении, кариесом и анкилостомозом и отрицательно связан с *Ascaris* и количеством вагинальной *Lactobacillus* и *Bacteroides/Gardnerella*. Более продолжительное вдыхание древесного дыма, кариес и повышенный уровень диплококковой инфекции увеличивали шансы иметь более высокую концентрацию CRP для каждого триместра, а более продолжительный гестационный срок снижал вероятность повышенных концентраций CRP для каждого триместра, что хорошо согласуется с приведёнными выше данными. Недостаточность фолиевой кислоты во время кормления была связана с повышенным CRP, а количество предыдущих родов, число эозинофилов и *Mobiluncus* были связаны с пониженным CRP. Кроме того, более высокий ИМТ и количество *Trichomonas vaginalis* увеличивали вероятность повышенного CRP, а большее количество предыдущих родов и эозинофилов были связаны с уменьшенной вероятностью повышенного CRP.

Выводы. Инфекции могут повышать и понижать концентрации CRP у беременных и кормящих матерей. Только недостаточность фолиевой кислоты во время кормления была связана с повышенными концентрациями CRP. Следует соблюдать осторожность при интерпретации концентраций CRP у беременных и кормящих женщин, которые при этом

страдают от недостаточности питательных веществ и множественных инфекций.

Translated from English version into Russian by Natalia Potashnik, through

TRANSLATORS
WITHOUT BORDERS

La proteína C Reactiva se ve significativamente modulada por infecciones concomitantes, deficiencias vitamínicas y factores maternales en mujeres indígenas de Panamá en período de embarazo o lactancia.

Doris González-Fernández, Emérita del Carmen Pons, Delfina Rueda, Odalis Teresa Sinisterra, Enrique Murillo, Marilyn E. Scott[6] y Kristine G. Koski

Resumen

Antecedentes: La utilidad de la proteína C reactiva (PCR) como marcador no-específico de inflamación durante el embarazo y la lactancia es poco claro en poblaciones empobrecidas donde son comunes las infecciones concomitantes y las deficiencias vitamínicas.

Métodos: El presente estudio transversal en Panamá reclutó a 120 mujeres Ngäbe-Buglé embarazadas y 99 en período de lactancia de 14 comunidades rurales de Panamá. Se midieron los antecedentes obstétricos, la exposición a humo de leña en espacios cerrados, trabajo en el campo, IMC, vitaminas A, B_{12}, D y ácido fólico y marcadores de inflamación (PCR, índice neutrófilo/linfocito (INL), plaquetocrito y citoquinas). Se utilizaron regresiones múltiples para explorar tanto asociaciones entre la PCR y otros marcadores de inflamación, como las asociaciones entre PCR y elevaciones de la PCR en base a límites trimestrales específicos con factores maternales, infecciones y deficiencias vitamínicas.

Resultados: La PCR se encontraba más elevada en el embarazo (51,4 ± 4,7 nmol/L) que durante la lactancia (27,8 ± 3,5 nmol/L) y se elevaba sobre los límites trimestrales específicos en el 21% de las mujeres embarazadas y 30% de las mujeres en lactancia. Las deficiencias vitamínicas fueron comunes (vitamina A 29,6%; vitamina D 68,5%; vitamina B_{12} 68%; ácido fólico 25,5%) y más del 50% de las mujeres presentaban dos o más deficiencias concomitantes e infecciones múltiples. Los modelos de regresión múltiple resaltaron las diferencias en las variables asociadas con PCR entre embarazo y lactancia. En el embarazo, la PCR se asociaba de manera positiva con una mayor exposición a humo de leña en espacios cerrados, caries y uncinariasis y se asociaba de manera negativa con *Ascaris* y *Lactobacillus* vaginal y puntajes de *Bacteroides/Gardnerella*. Consistente con lo anterior, una mayor exposición al humo de leña en espacios cerrados, la caries al igual que un puntaje más alto de infección por diplococos aumentaban las posibilidades de concentraciones trimestrales elevadas de PCR, mientras que una mayor edad gestacional disminuía las posibilidades de PCR trimestral elevada. Durante la lactancia, la deficiencia de ácido fólico se asociaba con una PCR más elevada mientras que la cantidad de partos, cantidad de eosinófilos y puntajes de *Mobiluncus* se asociaban con una PCR más baja. Asimismo, un mayor IMC y puntajes de *Trichomonas vaginalis* aumentaban las posibilidades de una PCR elevada mientras que una mayor

cantidad de partos y de eosinófilos se asociaban con una menor posibilidad de PCR elevada.
Conclusiones: Las infecciones aumentan y disminuyen las concentraciones de PCR en madres embarazadas y en lactancia. Solo la deficiencia de ácido fólico durante la lactancia se vio asociada con mayores concentraciones de PCR. Se debe proceder con cautela al interpretar las concentraciones de PCR en mujeres embarazadas y en período de lactancia que presentan deficiencias concomitantes de nutrientes e infecciones múltiples.

Translated from English version into Spanish by Maria Alejandra Aguada, through

TRANSLATORS
WITHOUT BORDERS
